# Unusual Temperature Dependence of Bandgap in 2D Inorganic Lead‐Halide Perovskite Nanoplatelets

**DOI:** 10.1002/advs.202100084

**Published:** 2021-08-11

**Authors:** Shaohua Yu, Jin Xu, Xiaoying Shang, En Ma, Fulin Lin, Wei Zheng, Datao Tu, Renfu Li, Xueyuan Chen

**Affiliations:** ^1^ CAS Key Laboratory of Design and Assembly of Functional Nanostructures State Key Laboratory of Structural Chemistry and Fujian Key Laboratory of Nanomaterials Fujian Institute of Research on the Structure of Matter Chinese Academy of Sciences Fuzhou Fujian 350002 China; ^2^ University of Chinese Academy of Sciences Beijing 100049 China; ^3^ Fujian Science and Technology Innovation Laboratory for Optoelectronic Information of China Fuzhou Fujian 350108 China; ^4^ Xiamen Institute of Rare Earth Materials Haixi Institute Chinese Academy of Sciences Xiamen Fujian 361021 China

**Keywords:** blueshift‐redshift crossover, inorganic lead‐halide perovskites, material dimensionality, temperature‐dependent bandgap

## Abstract

Understanding the origin of temperature‐dependent bandgap in inorganic lead‐halide perovskites is essential and important for their applications in photovoltaics and optoelectronics. Herein, it is found that the temperature dependence of bandgap in CsPbBr_3_ perovskites is variable with material dimensionality. In contrast to the monotonous redshift ordinarily observed in bulk‐like CsPbBr_3_ nanocrystals (NCs), the bandgap of 2D CsPbBr_3_ nanoplatelets (NPLs) exhibits an initial blueshift then redshift trend with decreasing temperature (290–10 K). The Bose–Einstein two‐oscillator modeling manifests that the blueshift‐redshift crossover of bandgap in the NPLs is attributed to the significantly larger weight of contribution from electron‐optical phonon interaction to the bandgap renormalization in the NPLs than in the NCs. These new findings may gain deep insights into the origin of bandgap shift with temperature for both fundamentals and applications of perovskite semiconductor materials.

## Graphical abstract

The temperature dependence of bandgap in inorganic lead‐halide perovskites is found to be variable with material dimensionality. In sharp contrast to the monotonous redshift usually observed in quasi‐3D bulk‐like CsPbBr_3_ nanocrystals (NCs), the bandgap of 2D 2‐monolayer‐thick (2‐ML‐thick) CsPbBr_3_ nanoplatelets (NPLs) exhibits an initial blueshift then redshift trend with decreasing temperature (290–10 K).

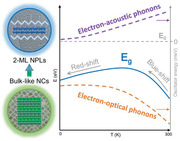

## 1 Introduction

Inorganic lead‐halide perovskites CsPbX_3_ (X = halide anion), with their dimensions varying from quasi‐3D bulk‐like nanocrystals (NCs) to 2D nanoplatelets (NPLs), 1D nanowires (NWs), and even to 0D quantum dots (QDs), have attracted intense interest of researchers because of their remarkable photovoltaic and optoelectronic performance.^[^ ^1^ ^]^ Particularly, 2D NPLs consisting of only a few atomic layers have recently aroused growing attention due to their most promising attributes such as high surface‐to‐volume ratio, strong quantum confinement, large exciton binding energy, reduced dielectric screening, giant oscillator strength, and superior conductivity in the lateral dimension, etc.^[^ ^2^ ^]^

For quasi‐3D bulk‐like CsPbX_3_ NCs, one ubiquitous peculiarity is that the bandgap exhibits a monotonous redshift with decreasing temperature, which is opposite to the blueshift of bandgap exhibited by most covalently bonded semiconductors (e.g., CdSe).^[^ ^3^ ^]^ The electronic bandgap is an important fundamental parameter of a semiconductor material in terms of its optical and optoelectric properties.^[^ ^4^ ^]^ The successes of CsPbX_3_‐based transistors, lasers, light‐emitting diodes, and solar cells have stimulated extensive theoretical and experimental studies on the temperature dependence of bandgap in CsPbX_3_ perovskites.^[^ ^5^ ^]^ From the theoretical point of view, the bandgap renormalization essentially arises from the lattice thermal expansion due to the anharmonicity of crystal potential, and from the electron‐phonon interactions.^[^ ^6^ ^]^ Recent investigations into CsPbX_3_ NCs on their bandgap redshift with decreasing temperature have been reported, and researchers tend to embrace the viewpoint of attributing the atypical redshift to the strong electron–phonon interactions in CsPbX_3_,^[^ ^7^ ^]^ whereas in‐depth insights into the underlying mechanism are still lacking. It is rationally expected that, concomitant with the reduction of material dimensionality of CsPbX_3_ from quasi‐3D to 2D or lower than 2D, the electron‐phonon interactions and consequently the bandgap renormalization would change accordingly in the low‐dimensional CsPbX_3_. Taking the 2D CsPbBr_3_ NPLs as an example, the electron structure and particularly the phonon structure such as the wave‐vector and the velocity of lattice wave in the quasi‐2D Brillouin zone for 2D CsPbBr_3_ NPLs differ from those in the quasi‐3D Brillouin zone for bulk‐like quasi‐3D CsPbBr_3_ NCs. Such a difference is due to the breaking translational periodicity in the thickness direction of 2D CsPbBr_3_ NPLs, the strong quantum confinement effect, and the reduced dielectric screening endowed by the low dielectric constant of surface organic ligands, etc. Thus, the changes in electron‐phonon interactions determined by the electron and phonon structures, and the consequent bandgap renormalization in CsPbBr_3_ NPLs relative to CsPbBr_3_ NCs are anticipated to occur.^[^ ^8^ ^]^

**Figure 1:**
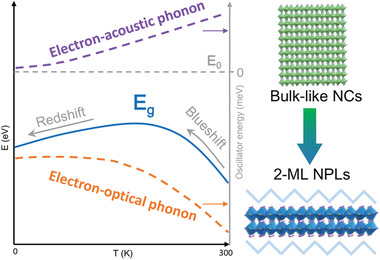
Schematic illustration of the unusual blueshift‐redshift crossover of bandgap with temperature in CsPbBr_3_ 2‐ML NPLs, which is tentatively attributed to the trade‐off between the opposite contributions of electron‐acoustic phonon and electron‐optical phonon interactions to the bandgap renormalization.

Herein, a comparative investigation on the temperature‐dependent bandgap in quasi‐3D bulk‐like CsPbBr_3_ NCs with weak quantum confinement and 2D 2‐monolayer‐thick (2‐ML‐thick) CsPbBr_3_ NPLs featuring strong quantum confinement was conducted. It is observed that, in contrast to the monotonous redshift of bandgap with decreasing temperature in CsPbBr_3_ NCs, the bandgap of CsPbBr_3_ 2‐ML NPLs displays an initial blueshift followed by redshift trend. The experimental investigations in combination with theoretical analyses indicate that such an unusual blueshift‐redshift crossover in the NPLs results from the trade‐off between the opposite contributions of electron‐acoustic phonon and electron‐optical phonon interactions to the bandgap renormalization (**Figure** **1**).

## 2 Results and Discussion

**Figure 2:**
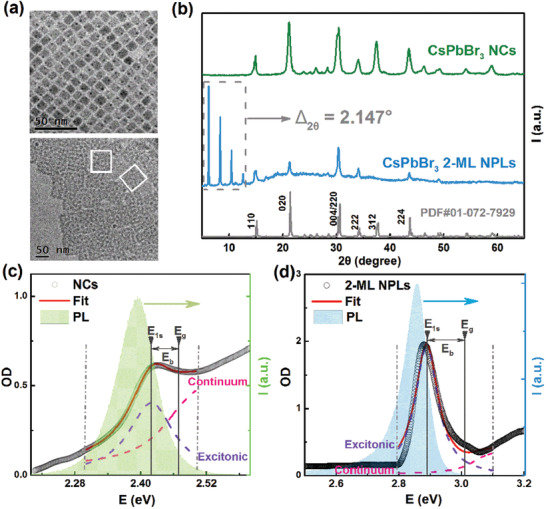
a) TEM images of CsPbBr_3_ NCs (upper) and 2‐ML NPLs (lower), respectively. b) XRD patterns of CsPbBr_3_ NCs and 2‐ML NPLs. Absorption (black circles) and PL emission spectra (shadow) of c) CsPbBr_3_ NCs and d) 2‐ML NPLs films recorded at 290 K, respectively. The fitting results based on Elliott's theory of Wannier excitons are plotted by red lines; the violet and pink dashed lines represent the contributions from 1s excitonic peak and the lowest band of continuum transition to the absorption near the band edge (area marked by grey dash‐dot line), respectively.

CsPbBr_3_ NCs and NPLs were synthesized following the previously reported experimental procedure,^[^ ^9^ ^]^ and their representative transmission electron microscopy (TEM) images were shown in **Figure** **2**a. The upper TEM image exhibits CsPbBr_3_ NCs with lateral sizes of around 10.6 × 8.5 nm, and the lower one with very low diffraction contrast signifies ultrathin NPLs of ≈72.5 × 68.9 nm. Figure 2b shows the X‐ray powder diffraction (XRD) patterns of CsPbBr_3_ NCs and NPLs, respectively, of which the peak positions match well with standard diffraction pattern of orthorhombic crystal structure.^[^ ^10^ ^]^ In comparison with the standard diffraction pattern, the slightly altered relative intensities of diffraction peaks for CsPbBr_3_ NCs reflect their cuboid‐like polyhedral morphologies, while the remarkably changed relative intensities for CsPbBr_3_ NPLs are indicative of the existence of preferred orientations of crystallographic planes.^[^ ^11^ ^]^ In addition to the (110) and (020) peaks, a series of strong equally spaced interplanar diffraction peaks (indicated by dashed rectangle in Figure 2b) resulting from the face‐to‐face stacking of the NPLs with high homogeneity in thickness were observed.^[^ ^12^ ^]^ The peak spacing of ≈ 2.147° indicates an average interplanar spacing (i.e., stacking distance) of ≈4.1 nm. Note that the interplanar spacing equals the NPLs thickness plus the two times length of the oleic acid ligand (≈1.5 nm) on each side of the single NPL, and the NPLs thickness can be approximated as n × 0.6 nm, where n represents the layer number of 2D arrangement of corner‐sharing [PbBr_6_]^4−^ octahedra with a thickness of ≈0.6 nm.^[^ ^13^ ^]^ Therefore, the measured stacking distance of ≈4.1 nm manifests an average NPL thickness of ≈1.1 nm, corresponding to approximately two monolayers of [PbBr_6_]^4−^ octahedra. Consistently, the TEM images showing stacked CsPbBr_3_ NPLs standing on their edges give an estimated thickness of a single NPL as ≈1.4 nm (Figure S1, Supporting Information).

The UV–vis absorption and photoluminescence (PL) spectra and absolute quantum yields (QYs) of CsPbBr_3_ NCs and 2‐ML NPLs were compared in Figures 2c,d and Table S1, Supporting Information. It can be seen that, contrary to the weak exciton absorption peak observed in CsPbBr_3_ NCs, the 2‐ML NPLs exhibit a sharp exciton resonance peak, verifying the strong quantum confinement in the NPLs.^[^ ^14^ ^]^ Furthermore, the absorption coefficient near the band edge was modeled by using Elliot's theory of Wannier excitons^[^ ^15^ ^]^ (Equations (8), (9a) in Section 4) to assign the contributions from the dominant 1s excitonic peak (violet dashed line) and the lowest band of the continuum transition (pink dashed line), respectively (Figures 2c,d). As such, we are able to extract the continuum absorption onset energy, that is, the bandgap energy *E*_g_, and also the position of 1s excitonic transition *E*_1s_. The exciton binding energy *E*_b_ was then determined by their difference: *E*_b_ = *E*_g_ − *E*_1s_.^[^ ^16^ ^]^ The extracted *E*_b_ for CsPbBr_3_ 2‐ML NPLs is ≈230 meV, a value nearly five times that for CsPbBr_3_ NCs (≈48 meV) (Figures 2c,d). Such a remarkable difference in *E*_b_ can be well explained when comparing the excitonic Bohr radius of bulk CsPbBr_3_ (≈3.5 nm)^[^ ^9^, ^17^ ^]^ with the NPL thickness (≈1.1 nm). Clearly, the 2‐ML NPLs of extremely large aspect ratio exhibit strong quantum confinement and possess reduced dielectric screening due to the surface passivation of oleic acid ligand with low dielectric constant, which consequently largely enhances the Coulomb interaction between electron and hole to form strongly bound exciton.^[^ ^2^, ^18^ ^]^ Correspondingly, owing to the strong quantum confinement in the CsPbBr_3_ 2‐ML NPLs, their PL peak shows a large blueshift relative to that of bulk‐like CsPbBr_3_ NCs with weak quantum confinement (Figures 2c,d).^[^ ^19^ ^]^ Moreover, the 2‐ML NPLs display a longer PL tail than the NCs, in consistency with the result reported by Bohn et al.^[^ ^20^ ^]^ Such a long low‐energy PL tail in the 2‐ML NPLs is rationally attributed to the radiative recombination of trap states^[^ ^21^ ^]^ when considering that the inhomogeneity in thickness distribution of the NPLs is excluded (Figure S2, Supporting Information).

**Figure 3:**
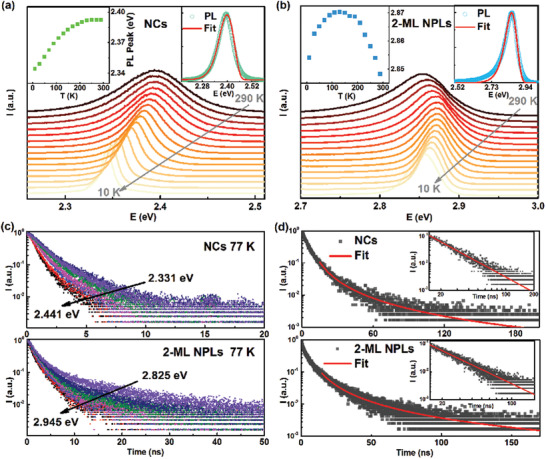
a) Normalized PL emission spectra a) of CsPbBr_3_ NCs and b) 2‐ML NPLs films measured over the temperature range of 10–290 K under excitation at 365 nm. Insets: PL peak energy position versus temperature (left) and fit of PL emission spectrum at 290 K by line‐shape function (right). c) Wavelength‐dependent PL lifetimes of CsPbBr_3_ NCs (upper) and 2‐ML NPLs films (lower) at 77 K with the 375‐nm ps‐pulsed laser as the excitation source. d) PL peak decay curves of the CsPbBr_3_ NCs (upper) and 2‐ML NPLs (lower) films at 290 K and their corresponding fits by power‐law model, displayed in the semi‐logarithmic scale. Inset: the enlarged fitted decay curve spanning over the middle to caudal segment in the double logarithmic scale.

The differences in optical properties between CsPbBr_3_ NCs and 2‐ML NPLs were further investigated by measuring their PL spectra over the temperature range of 10‐290 K (**Figures** **3**a,b). Each spectrum was fitted by using a Lorentzian curve, enabling the extraction of the PL peak position versus temperature (left insets of Figures 3a,b). For the CsPbBr_3_ NCs, their PL peak shows a redshift as the temperature decreases from 290 to 10 K (Figure 3a). By contrast, a trend of blueshift followed by redshift was observed for the CsPbBr_3_ 2‐ML NPLs (Figure 3b). Given that the PL peak position is to a large extent dictated by the *E*_g_ value, the change of PL peak position is generally regarded as equal to the shift of bandgap, as reported in the literature.^[^ ^6^, ^22^ ^]^ However, owing to the ubiquitous existence of radiative trap states (i.e., localized charge‐carriers) in semiconductor nanomaterials, there exists a deviation to some extent between the change of PL peak position and the shift of bandgap. As a matter of fact, the radiative recombination of trap states is also part of what constitutes the PL spectrum, besides the emission of free excitons.^[^ ^23^ ^]^ To illustrate this point, the semi‐empirical line‐shape function, developed initially for analyzing the exponential PL tail of Si and then widely applied in II–VI colloidal QDs, was introduced to simulate the PL spectra of CsPbBr_3_ NCs and 2‐ML NPLs at 290 K, respectively:

(1) $$\begin{array}{ccc} I\left(E,T\right) & = & Ae^{-\left(E_{g}-E\right)/W_{d}}\cdot {\left[1-e^{-\left(E_{g}-E\right)/W_{d}}\right]}^{N}\\ & & \cdot {\left[1+\frac{M}{e^{\left(E_{a}-E\right)/K_{b}T}-1}\right]}^{-1} \end{array}$$

where *E*_a_ represents the effective position of energy barrier for the thermal quenching of excited states, *M* is the intrinsic detrapping rate divided by the radiative rate of emitting state, *N* is related to the density of trap states, and *W*_d_ is the width of the exponential PL tail.^[^ ^24^ ^]^ It is worth mentioning that the semi‐empirical line‐shape function merely provides a phenomenological interpretation for the experimental data, thus is not appropriate to be used for the accurate determination of *E*_g_. The PL spectra of CsPbBr_3_ NCs and 2‐ML NPLs were well fitted by the line‐shape function, except the small deviation at low‐energy tail of PL spectrum for the NPLs (right insets of Figures 3a,b, Table S1, Supporting Information). Such a deviation at PL tail in turn indicates that the radiative recombination of trap states possesses a more complicated mathematical form in the actual situation. The impact of trap states on the PL properties was further reflected in the wavelength‐dependent PL lifetimes of CsPbBr_3_ NCs or 2‐ML NPLs. To be specific, the lifetime decreases rapidly with an increase in the energy of PL (Figures 3c, Figure S3, S4, Supporting Information), substantially attributed to the energy relaxation from the free excitons to the localized low‐lying trap states.^[^ ^21^, ^25^ ^]^ The distribution density of low‐lying trap states can be roughly evaluated by estimating the value of characteristic energy depth to band edge through fitting the decay curve of PL peak by the modified power‐law decay model: *I*(t) ∝ $t^{-(1+K_{b}T/\varepsilon_{0})}$ (*K*_b_ is the Boltzmann constant) (Equations (10), (11), (12), (13), (14), (15), (16) in Section 4), where *ε*_0_ represents the characteristic energy depth, and the ratio of trap states lying above *ε*_0_ equals (1 ‐ 1/*e*) ≈ 63% (Figure S5, Supporting Information).^[^ ^21b^ ^]^ Note that the modified power‐law decay model is applicable to simulating the segment of decay curve wherein *v*_0_ *t* ≫1 (*ν*_0_ is the attempt‐to‐escape frequency) is satisfied (Equations (15), (16) in Section 4). Figure 3d shows the best fit of the middle to caudal segment of decay curve, giving characteristic depth *ε*_0_ ≈ 28 and 35 meV for the CsPbBr_3_ NCs and 2‐ML NPLs, respectively. The larger value of *ε*_0_ for the 2‐ML NPLs denotes that there is a broader density distribution of trap states in the NPLs than in the NCs. All in all, for the CsPbBr_3_ NCs, especially for the 2‐ML NPLs, the change of their PL peak with temperature is also partially attributed to the variation in the temperature‐dependent radiative recombination probability and density of trap states, in addition to its association with the shift of *E*_g_. Thus, the more or less deviation between the measured change of PL peak position and the actual shift of *E*_g_ would impose ambiguity for the investigation on the temperature‐dependent bandgap.

**Figure 4:**
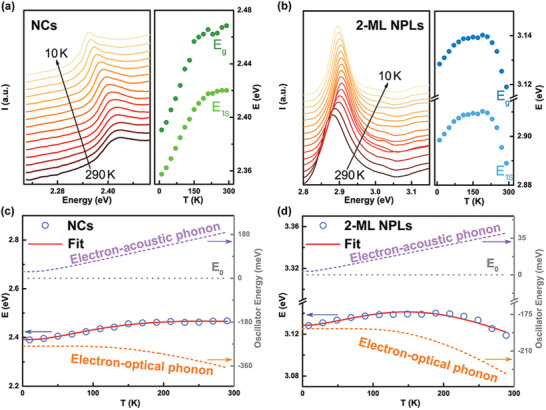
a–b) Absorption spectra (left) of a) CsPbBr_3_ NCs and b) 2‐ML NPLs films, respectively, measured over the temperature range of 10–290 K with a temperature interval of 20 K, and the corresponding *E*_g_ and *E*_1s_ values (right) extracted through fitting the absorption spectra by Elliot model. c,d) Bandgap energy of c) CsPbBr_3_ NCs and d) 2‐ML NPLs as a function of temperature from 10 to 290 K (blue circle), and their corresponding fits by the Bose–Einstein two‐oscillator model (red line), consisting of the contributions of electron‐acoustic phonon (violet dashed line) and electron‐optical phonon (orange dashed line) interactions to the bandgap shift with temperature. The horizontal grey dotted line denotes the unrenormalized bandgap energy *E*_0_.

For the purpose of more accurate determination of the bandgap shift with temperature, the temperature‐dependent absorption spectra of CsPbBr_3_ NCs and 2‐ML NPLs were monitored, respectively. The extracted *E*_g_ and *E*_1s_ values through fitting the absorption coefficient near the band edge based on Elliot model (Equations (8), (9a) in the Section 4) were plotted in **Figures** **4**a,b, with fitting parameters listed in Tables S2, S3, Supporting Information. Obviously, the shift in *E*_g_ with decreasing temperature differs from that in PL peak position at each temperature point for both the NCs and NPLs, yet they exhibit similar variation trends (right halves of Figures 4a,b, left insets of Figures 3a,b). The *E*_g_ of CsPbBr_3_ NCs shows a monotonous redshift trend with decreasing temperature, while that of 2‐ML NPLs displays a trend of initial blueshift followed by redshift (right halves of Figures 4a,b). In contrast to the widely observed redshift of bandgap in CsPbBr_3_ NCs,^[^ ^3^, ^7^, ^26^ ^]^ such an unusual blueshift‐redshift crossover in the 2‐ML NPLs had never been reported before.

Over the past few years, the empirical Varshni model^[^ ^27^ ^]^ and the semi‐empirical Bose–Einstein oscillator model^[^ ^28^ ^]^ have been widely used to describe the observed bandgap shift with temperature (*E*_g_(*T*)) in semiconductor materials. The empirical Varshni model reads: $E_{g}\left(T\right)=E_{0}-\alpha T^{2}/\left(\beta +T\right)$, where E_0_ is the value of bandgap at 0 K, *α* is a constant and *β* is related to the Debye temperature. In the high temperature regime, Varshni model predicts a linear relationship between the bandgap and temperature, which is consistent with the observation in many IV, III–V and II–VI semiconductors.^[^ ^29^ ^]^ However, in the low temperature regime, the predicted quadratic dependence by Varshni model contradicts with the temperature independence observed in some semiconductors at very low temperatures.^[^ ^28^, ^30^ ^]^ In this work, the CsPbBr_3_ 2‐ML NPLs exhibited a non‐monotonous initial blueshift followed by redshift trend of bandgap with decreasing temperature (290‐10 K). Note that the first derivative of Varshni model reads: $dE_{g}\left(T\right)/dT=-\alpha \cdot \left(2\beta T+T^{2}\right)/{\left(T+\beta \right)}^{2}$. Therefore, the Varshni model can describe a monotonous blueshift or redshift trend, but fails to simulate a non‐monotonous variation trend, such as the blueshift‐redshift crossover observed in the CsPbBr_3_ 2‐ML NPLs. In theory, under the quasi‐harmonic approximation, the derivative of bandgap with temperature can be expressed by:

(2) $$\frac{dE_{g}}{dT}={\left(\frac{\partial E_{g}}{\partial T}\right)}_{\mathrm{TE}}+\sum_{j,\vec{q}}\frac{\partial E_{g}}{\partial n_{j,\vec{q}}}\left(n_{j,\vec{q}}+1/2\right)$$

The first term in Equation 2 accounts for the lattice thermal expansion (TE) contribution to the bandgap shift. The second component corresponds to the bandgap renormalization caused by electron–phonon interactions: $n_{j,\vec{q}}$ is the Bose‐Einstein phonon occupation factor at j branch with wave vector $\vec{q}$, and the real part of the complex electron‐phonon interaction coefficient $\partial E_{g}/\partial n_{j,\vec{q}}$ contributes to the bandgap shift.^[^ ^31^ ^]^ It is noteworthy that CsPbBr_3_ is in orthorhombic phase below ≈380 K; the lattice transforms to tetragonal phase through octahedral tilting at ≈380 K, and becomes cubic at ≈403 K.^[^ ^10d^ ^]^ Thus, no phase transition from the orthorhombic to tetragonal or cubic phase occurred for CsPbBr_3_ perovskites in the temperature range concerned in this work (10–290 K).

The thermal expansion contribution is usually estimated by the product of the volumetric expansion coefficient *α*_*ν*_, the bulk modulus *B*_0_, and the pressure coefficient of bandgap *dE*_g_/*dP*:

(3) $${\left(\frac{\partial E_{g}}{\partial T}\right)}_{\mathrm{TE}}=\frac{dE_{g}}{dV}\cdot \frac{dV}{dT}=-\frac{dV}{dT}\cdot \left|\frac{dP}{dV}\right|\cdot \frac{dE_{g}}{dP}=-\alpha_{v}\cdot B_{0}\cdot \frac{dE_{g}}{dP}$$

where the value of *dE*_g_/*dP* can be experimentally determined through the high pressure experiments.^[^ ^31^, ^32^ ^]^ On the one hand, it should be noted that the lattice can never be frozen, even at 0 K because of the zero‐point vibration energy.^[^ ^33^ ^]^ On the other hand, It is well accepted that lattice vibrations play a central role in electron‐phonon interactions, and phonon frequencies are dependent on the equilibrium volume of crystal lattice.^[^ ^34^ ^]^ In other words, electron–phonon interactions rely on the equilibrium volumes of crystal lattice at different pressures in the high pressure experiments. As a result, the thermal expansion contribution is overestimated because the measured value of *dE*_g_/*dP* inevitably incorporates the contribution of electron–phonon interactions due to the dependence of the harmonic frequencies of lattice on structural changes in the high pressure experiments. However, this approximate calculation method still provides an experimental approach to investigate solely the bandgap change with increasing or decreasing lattice constant.

In the literature, for a large variety of semiconductor materials and in particular the lead‐based compounds, the thermal expansion contribution to bandgap renormalization was not taken into account because it had a relatively small magnitude with respect to the contribution from electron–phonon interactions.^[^ ^35^ ^]^ For instance, when discussing the bandgap renormalization of CuCl^[^ ^36^ ^]^ and AgGaS_2_,^[^ ^37^ ^]^ the researchers only considered the contribution from electron‐phonon interactions by adopting the Bose–Einstein oscillator model (vide infra) to fit the bandgap shift with temperature. Also, for lead‐halide perovskites, Tilchin et al.,^[^ ^35^ ^]^ and Saran et al.,^[^ ^7^ ^]^ etc. attributed the bandgap shift of lead‐halide perovskites featuring corner‐sharing [PbBr_6_]^4−^ octahedra to the electron‐phonon interactions. Theoretically, the lattice thermal expansion effect can be accounted for by calculating the band structure of the system as a function of the equilibrium lattice constant at different temperatures. Specifically, one can treat this problem by freezing the lattice (i.e., under adiabatic condition), and then calculate the band structure as the lattice constant rigidly varies from a_0_ to a_0_‐Δa, where *a*_0_ is the lattice constant at room temperature. For example, based on the density functional theory calculations using the equilibrium lattice parameters of the tetragonal phase of MAPbI_3_ (MA=CH_3_NH_3_) single crystals at different temperatures, Saidi et al. found that the contribution from lattice expansion to the bandgap shift was an order of magnitude smaller than that from electron–phonon interactions.^[^ ^38^ ^]^ More importantly, Saidi et al. reported in another paper that the low‐energy phonon modes of corner‐sharing [PbI_6_]^4−^ octahedra dominated such bandgap shift related electron–phonon interactions over the high‐energy modes of organic cation in MAPbI_3_ single crystals.^[^ ^39^ ^]^ This is consistent with the fact that the conduction band minimum (CBM) and valence band maximum (VBM) of MAPbI_3_ are formed by states whose characters are dominated by the p‐band of Pb, the s‐band of Pb and p‐band of iodine in the [PbI_6_]^4−^ octahedra, respectively.^[^ ^40^ ^]^ Thereafter, following Saidi et al.’s study, researchers recently adopted the viewpoint of attributing the bandgap shift of lead‐halide perovskites featuring corner‐sharing [PbX_6_]^4−^ octahedra exclusively to the electron‐phonon interactions.^[^ ^41^ ^]^ In this context, the lattice thermal expansion contribution to the temperature dependence of bandgap in CsPbBr_3_ perovskites was not taken into account in this work.

According to Fan's model based on the 2nd‐order perturbation theory,^[^ ^42^ ^]^ the contribution of electron‐phonon interactions to the bandgap shift Δ*E*_g_ is given by:^[^ ^43^ ^]^

(4) $$\begin{array}{ccc} \Delta E_{g} & = & -\sum_{\vec{q}}\frac{{\left|\langle \vec{k}\left|H_{\mathrm{el}-\mathrm{ph}}\right|\vec{k}-\vec{q}\rangle \right|}^{2}}{E\left(\vec{k}-\vec{q}\right)-E\left(k\right)+\hbar \omega_{\vec{q}}}\\ & & -\;\sum_{\vec{q}}\frac{{\left|\langle \vec{k}\left|H_{\mathrm{el}-\mathrm{ph}}\right|\vec{k}+\vec{q}\rangle \right|}^{2}}{E\left(\vec{k}+\vec{q}\right)-E\left(k\right)-\hbar \omega_{\vec{q}}} \end{array}$$

where the first and second terms correspond to the emission and absorption of phonons at wave‐vector $\vec{q}$, respectively, $\vec{k}$ is the wave‐vector of electron, $\omega_{\vec{q}}$ is the angular frequency of phonon mode $\vec{q}$, *H*_el‐ph_ is the Hamiltonian of electron–phonon interactions. For the acoustic phonons, if only considering the wave‐vector zone of $\vec{k}\approx 0$, then,

(5) $$\Delta E_{g}=-A\sum_{\vec{q}}\frac{4m^{*}}{{\left|\vec{q}\right|}^{2}-Q^{2}}\cdot \frac{1}{e^{\hbar \omega_{\vec{q}}/K_{b}T}-1}$$

where *A* is a positive constant, m* is the effective mass of carriers, and $Q=\left|2m^{*}v_{\vec{q}}/\hbar \right|$, with $v_{\vec{q}}$ denoting the velocity of acoustic wave near the Brillouin zone edge. For the optical phonons, the situation is quite similar to the acoustic phonons. Meanwhile, for simplicity, if only considering longitudinal optical phonon modes and assuming very flat dispersion for these modes, then,

(6) $$\Delta E_{g}=-B\sum_{\vec{q}}\frac{m^{*}{\left|\vec{q}\right|}^{2}}{{\left|\vec{q}\right|}^{4}-P^{4}}\cdot \frac{1}{e^{\hbar \omega_{\mathrm{LO}}/K_{b}T}-1}$$

where *B* is a positive constant, ℏ*ω*_LO_ is a characteristic longitudinal optical phonon energy and $P=\sqrt{2m^{*}\hbar \omega_{\mathrm{LO}}}/\hbar$.

In practice, however, it remains challenging to directly calculate the electron‐phonon interaction term (Equation 4) which requires summation extending all over photon modes. In order to circumvent this obstruction, efforts have been made to approximate the electron‐phonon interaction term by employing electron‐phonon interaction parameter *A*_i_ for phonon with effective phonon energy *E*_i_. Under this approximation, Equations 5 and 6 can lead to the Bose–Einstein oscillator model: $\sum_{i}\left(A_{i}/M_{i}E_{i}\right)\left(1/e^{E_{i}/K_{b}T}+1/2\right)$, where *M*_i_ is the atomic mass of oscillator.^[^ ^31a^ ^]^ In this work, a Bose–Einstein two‐oscillator model was adopted to analyze the temperature dependence of bandgap in CsPbBr_3_ perovskites, considering that Pb and Br atoms have markedly different masses like in the case of the cuprous halide semiconductors.^[^ ^31a^ ^]^ Accordingly, the temperature‐dependent *E*_g_ can be expressed by:

(7) $$\begin{array}{ccc} E_{g}\left(T\right) & = & E_{0}+\frac{A_{\mathrm{ac}}}{M_{\mathrm{ac}}E_{\mathrm{ac}}}\left(\frac{1}{e^{E_{\mathrm{ac}}/K_{b}T}-1}+\frac{1}{2}\right)\\ & & +\;\frac{A_{\mathrm{opt}}}{M_{\mathrm{opt}}E_{\mathrm{opt}}}\left(\frac{1}{e^{E_{\mathrm{opt}}/K_{b}T}-1}+\frac{1}{2}\right) \end{array}$$

where *E*_0_ is the unrenormalized bandgap energy, *A*_ac_ (*A*_opt_) represents the electron‐phonon interaction coefficient for effective acoustic (optical) phonon energy *E*_ac_ (*E*_opt_) with oscillator atomic mass *M*_ac_ (*M*_opt_).^[^ ^36^, ^37^ ^]^ It is clear that the electron‐phonon interaction coefficient *A*_ac_ or *A*_opt_ is determined by both the electron and phonon structures, which can be exemplified by the series of coefficients $4m^{*}/\left({|\vec{q}|}^{2}-Q^{2}\right)$ for acoustic phonons in Equation 5 or $m^{*}|\vec{q}{|}^{2}/\left({|\vec{q}|}^{4}-P^{4}\right)$ for optical phonons in Equation 6. Previous studies in the literature revealed that the lack of translational periodicity in low‐dimensional semiconductor nanomaterials affected both the phonon structure and the electron‐phonon interactions.^[^ ^8b^ ^]^ Krauss et al. observed that the breaking translational symmetry in PbS QDs caused mixing of the transverse and longitudinal optical phonon modes.^[^ ^44^ ^]^ Itoh et al. reported that the electron‐phonon interactions in CuCl QDs could be significantly enhanced, which eventually resulted in a frequency renormalization of the longitudinal optical mode.^[^ ^45^ ^]^ Furthermore, the reduced dielectric screening of carriers in 2D MAPbI_3_ NPLs due to the low dielectric constant of organic ligands surrounding the NPLs was reported to leading to high electron–optical phonon scattering rates.^[^ ^8^, ^46^ ^]^ For CsPbBr_3_ perovskites with dimensions varying from quasi‐3D bulk‐like NCs to 2D NPLs, 1D NWs, and to 0D QDs, their Brillouin zone, wave‐vectors $\vec{q}$, velocity of acoustic wave $v_{\vec{q}}$, and consequently the electron–phonon interactions differ from each other to some extent. However, the above differences can be taken into account by calculating the electron‐phonon interaction term through summation extending all over photon modes in Equation 4. Thus, the differences in the contributions of electron–phonon interactions to the bandgap renormalization between CsPbBr_3_ perovskites with varying dimensions are finally reflected in the distinct values of electron–phonon interaction parameter *A*_i_, effective phonon energy *E*_i_ in Equation 7. As a result, Equation 7 is valid for lead‐halide perovskites with different dimensions ranging from 3D to 0D. Herein, for the 2D CsPbBr_3_ 2‐ML NPLs with breaking translational periodicity in the thickness direction, it is rationally expected that the electron structure and particularly the phonon structure in the quasi‐2D Brillouin zone, for instance, the wave‐vector $\vec{q}$ and the velocity of acoustic wave $v_{\vec{q}}$ in Equations (5), (6), differ to some extent from those in the quasi‐3D Brillouin zone for bulk‐like quasi‐3D CsPbBr_3_ NCs. Also, the strong quantum confinement effect and the reduced dielectric screening due to the low dielectric constant of surface organic ligands in CsPbBr_3_ 2‐ML NPLs can influence the carrier parameters such as the effective mass m* in Equations (5), (6). Therefore, the changes in electron‐phonon interactions and consequently the temperature dependence of bandgap in CsPbBr_3_ 2‐ML NPLs relative to CsPbBr_3_ NCs are anticipated.^[^ ^8b^ ^]^

To qualitatively evaluate the changes in electron‐phonon interactions for 2D CsPbBr_3_ 2‐ML NPLs as compared to bulk‐like quasi‐3D CsPbBr_3_ NCs, the Bose‐Einstein two‐oscillator model was adopted to estimate the effective electron–phonon interaction coefficient through fitting the temperature‐dependent bandgap. As compared in Figures 4c,d, the Bose‐Einstein two‐oscillator model gives an excellent fit (red line) to the *E*_g_ extracted from absorption spectra for both the CsPbBr_3_ NCs and 2‐ML NPLs over the temperature range investigated, with fitting parameters presented in Table S4. The derived effective acoustic (optical) phonon energies are ≈6.9 meV (≈48.1 meV) for the NCs and ≈4.0 meV (≈55.0 meV) for the 2‐ML NPLs, respectively (Table S4, Supporting Information). The large difference between acoustic and optical phonon energies validates that the acoustic phonons are dominated by the displacement of heavy Pb atoms, and the optical phonons by the displacement of light halogen atoms.^[^ ^7^ ^]^ It should be mentioned that the fitting results in Figures 4c,d represent the screened optimal solution of Equation 7 for the fitting of temperature‐dependent *E*_g_ of CsPbBr_3_ NCs and 2‐ML NPLs, when comprehensively considering the correlation coefficient of fit, *R* ^2^, and the aspect of physical meaning of fitting parameters. For instance, as shown in Figure S6, Supporting Information, for the case of CsPbBr_3_ 2‐ML NPLs, if the fitting parameter *E*_opt_ (i.e., effective optical phonon energy) was fixed at ≈18 meV, a value that was in the same order of magnitude as the maximum cut‐off phonon energy (≈16 meV) of CsPbBr_3_ crystal lattice, only a poor fit (Fit 2 with *R* ^2^ = 0.685) to the temperature‐dependent *E*_g_ was acquired. Additionally, Fit 3 shows the best fit with *R* ^2^ = 0.964, but lacks physical meaning, because the fitting parameter *E*_0_ (i.e., unrenormalized bandgap energy) is as large as 4.264 eV, which is approximately 1.1 eV larger than the *E*_g_ value of 3.119 eV for CsPbBr_3_ 2‐ML NPLs at 290 K (Figure S6, Supporting Information).

As listed in Table S4, Supporting Information, the derived optical phonon energies for CsPbBr_3_ NCs and 2‐ML NPLs (in the order of 50 meV) are much larger than the maximum cut‐off phonon energy of CsPbBr_3_ crystal lattice (≈16 meV) (Figure S7, Supporting Information). Such a contradiction can be illustrated by comparing the mathematic forms of Fan's and the Bose‐Einstein oscillator models. Specifically, if only considering the electron wave‐vector zone of $\vec{k}\approx 0$, the electron‐optical phonon interaction term of Fan's model can be expressed as: $-B\sum_{\vec{q}}\frac{m^{*}{\left|\vec{q}\right|}^{2}}{{\left|\vec{q}\right|}^{4}-P^{4}}\cdot \frac{1}{e^{\hbar \omega_{\mathrm{LO}}/K_{b}T}-1}$ (Equation 6) based on some approximations, which is equivalent to the Bose‐Einstein single‐oscillator model: $\frac{A_{\mathrm{opt}}}{M_{\mathrm{opt}}E_{\mathrm{opt}}}\left(\frac{1}{e^{E_{\mathrm{opt}}/K_{b}T}-1}+\frac{1}{2}\right)$. Apparently, the derived effective optical phonon energy (*E*_opt_) is constrained by both the series of coefficients $m^{*}|\vec{q}{|}^{2}/\left({|\vec{q}|}^{4}-P^{4}\right)$ and the characteristic longitudinal optical phonon energy ℏ*ω*_LO_, i.e., *E*_opt_ ≠ ℏ*ω*_LO_. Basically, the direct calculation of the electron–phonon interaction term of Fan's model requires summation extending all over photon modes in the Brillouin zone, thus the large difference between the derived effective optical phonon energy and the maximum cut‐off phonon energy of CsPbBr_3_ crystal lattice implies the extreme complexity of the electron‐phonon interaction coefficient $\partial E_{g}/\partial n_{j,\vec{q}}$ for the phonon mode at j branch with wave vector $\vec{q}$ (Equation 2).

The fitting results in Table S4, Supporting Information exhibited that the effective electron‐acoustic phonon interaction coefficients *A*_ac_ / (*M*_ac_ *E*_ac_) dropped remarkably from ≈ 0.054 eV for CsPbBr_3_ NCs to ≈0.007 eV for CsPbBr_3_ 2‐ML NPLs. The effective electron‐optical phonon interaction coefficients *A*_opt_ /(*M*_opt_ *E*_opt_) increased mildly from ≈−0.557 eV for the NCs to ≈−0.377 eV for the 2‐ML NPLs. Consistently, the electron‐optical phonon interaction coefficient (*A*_LO_) and the effective energy of optical phonon (*E*_LO_), derived from fitting the temperature‐dependent full width at half maximum for CsPbBr_3_ 2‐ML NPLs by adopting Segall's expression,^[^ ^47^ ^]^ are much larger than those for CsPbBr_3_ NCs (Table S5 and Figure S8, Supporting Information). Therefore, it can be concluded that the more enhanced weight of contribution of electron‐optical phonon interaction in CsPbBr_3_ 2‐ML NPLs than in CsPbBr_3_ NCs gives rise to the observed blueshift‐redshift crossover of bandgap with decreasing temperature in the NPLs (Figure 4d).

For perovskite CsPbBr_3_, it is important to note that: 1) the valence band maximum (VBM) of CsPbBr_3_ consists of s‐band of Pb and p‐band of Br with antibonding interaction between them, while the conduction band is formed of p‐band of Pb;^[^ ^48^ ^]^ 2) the crystal structure of CsPbBr_3_ is composed of a framework of corner‐sharing [PbBr_6_]^4−^ octahedra, with A‐site metal cations Cs^+^ occupying cubo‐octahedral voids in between. Consequently, we speculate that the bandgap renormalization in CsPbBr_3_ is closely related to the antibonding properties of the 6s^2^ lone pair of Pb coupled to Br 4p orbital, as a factor directly influencing the electron‐phonon scattering in the corner‐sharing [PbBr_6_]^4−^ framework. It is worth mentioning that, similar to CsPbX_3_ perovskites, PbS or PbSe semiconductor materials usually exhibit a redshift of bandgap with decreasing temperature.^[^ ^49^ ^]^ Zunger et al.’s work showed that this anomaly in PbS resulted from the occurrence of the filled Pb s‐band below the top of the valence band, setting up coupling and level repulsion at the L point in the Brillouin zone.^[^ ^50^ ^]^ Furthermore, recent size‐dependent studies on PbS or PbSe revealed that for small‐sized QDs (e.g., *d* ≈ 2.8 nm) such a redshift behavior disappeared, and in the case of QDs with further smaller size, the bandgap even displayed a blueshift with decreasing temperature.^[^ ^51^ ^]^ Additionally, some recently reported representative examples of CsPbBr_3_ perovskites exhibiting distinct variation trends of spontaneous emission (or lasing, reflectance) spectra were summarized in **Table** **1**. For instance, in Li et al.’s work,^[^ ^21a^ ^]^ the PL spectra of ultrasmall CsPbBr_3_ QDs with an average diameter of ≈2.7 nm presented a monotonous blueshift trend with decreasing temperature in the range of 225–19 K, contrary to the redshift usually observed in bulk‐like CsPbBr_3_ NCs. Also, Ai et al.^[^ ^52^ ^]^ also studied the PL properties of CsPbBr_3_ QDs embedded in glasses, and they found that the CsPbBr_3_ QDs with average radii of ≈4.8 nm showed a redshift trend of PL peak with decreasing temperature (240‐40 K), while the CsPbBr_3_ QDs with average radii of ≈3.3 nm exhibited an initial blueshift followed by redshift trend. By the way, Liu et al.^[^ ^53^ ^]^ reported that the energies of lasing peaks of CsPbBr_3_ nanowires exhibited a blueshift‐redshift crossover with decreasing temperature (295‐78 K), whereas the energies of spontaneous emission peaks of CsPbBr_3_ nanowires still presented a monotonous redshift trend (Table 1). In Liu et al.’s work, although the underlying reasons for the discrepancy between the variation trends of spontaneous emission and lasing spectra of CsPbBr_3_ nanowires was not elaborated by the authors, such markedly different variation trends at least demonstrated that the bandgap renormalization was not the sole determinant of the shift of lasing spectra with temperature.

**Table 1:** Summary of the recently reported variation trends of spontaneous emission (or lasing, reflectance) spectra in CsPbBr_3_ perovskites with dimensions ranging from 3D bulk crystals, quasi‐3D bulk‐like NCs to 2D NPLs, 1D NWs, and to 0D QDs

Dimension	Size	Variation trend with decreasing temperature	Ref.
bulk crystals	–	Redshift (300–10 K)	[56]
NCs	15 nm (edge length)	Redshift (300–3 K) (reflectance spectrum)	[3a]
NCs	11 nm (edge length)	Roughly constant (380–220 K)‐redshift (220–80 K)	[57]
QDs	1.3 nm (radius)	Blueshift (225–19 K)	[21a]
QDs embedded in phosphate glasses	3.3 nm 4.2 nm 4.8 nm (radius)	Blueshift (240–140 K)‐redshift (140–40 K) Blueshift (240–180 K)‐redshift (180–40 K) Redshift (240–40 K)	[52]
NWs	12 nm × 5 µm (diameter × length)	Redshift (295–5.8 K)	[58]
NWs	372 nm × 22 µm (width × length)	Redshift (295–78 K) (spontaneous emission peak) Blueshift (295–195K)‐redshift (195–78 K) (lasing peak)	[53]
Microplatelets	150 nm × 7–15 µm (thickness × lateral size)	Redshift (300–10 K)	[3c]
NPLs	3.4 nm × 18.2 nm (thickness × lateral size)	Redshift (300–16 K)	[59]

In analogy with the layered structure of CsPbBr_3_ 2‐ML NPLs, the recently widely reported 2D layered perovskites (such as 2D Ruddlesden‐Popper perovskites), featuring metal halide slabs separated by the organic layers with a dielectric constant that is smaller than that of the inorganic layer, are naturally formed multiple quantum‐well (QW) materials.^[^ ^54^ ^]^ In 2D perovskites, it has been demonstrated that the band gap shows a quite different behavior with temperature depending on the layer number n.^[^ ^55^ ^]^ For instance, Li et al. found that, in (n‐BA)_2_(MA)_n−1_PbnI_3n+1_ ((BA = C_4_H_9_NH_3_, and MA = CH_3_NH_3_) microplates, the emission peak exhibited first a blueshift and then redshift for *n* = 2 or 3 samples with an increase in temperature from 77 to 290 K, while a monotonic blueshift was observed for *n* = 4 and *n* = 5 samples, which is similar to the emission peak shift in the case of the 3D perovskites.^[^ ^6^ ^]^ Particularly, for the *n* = 2 or 3 (n‐BA)_2_(MA)_n−1_Pb_n_I_3n+1_ samples, owing to the breaking translational periodicity in the thickness direction of the 2D corner‐sharing [PbI_6_]^4−^ slabs, the strong quantum confinement effect, and the reduced dielectric screening due to the low dielectric constant of organic ligands n‐BA, the phonon structures and consequently the bandgap renormalization deriving from electron‐phonon interactions are expected to change remarkably relative to the *n* = ∞ 3D perovskites counterparts. In this regard, the analogous observation that layer number n affects the temperature evolution of the bandgap for 2D perovskites, in turn, further support our viewpoint that the unusual temperature dependence of bandgap in 2D 2‐monolayer‐thick (2‐ML‐thick) CsPbBr_3_ NPLs is dominantly attributed to the trade‐off of optical and acoustic phonon‐electron scattering. All in all, the above findings reported in the literature exemplify that the NC size, or more exactly the material dimensionality reveals close relevance with the temperature dependence of bandgap in Pb‐containing semiconductor materials.

## 3 Conclusion

In summary, a comprehensive survey has been performed on the temperature dependence of bandgap in quasi‐3D bulk‐like CsPbBr_3_ NCs and 2D 2‐ML‐thick CsPbBr_3_ NPLs by monitoring their PL and absorption spectra over the temperature range of 10–290 K. Particularly, it is revealed that there exists an unavoidable deviation between the change of PL peak position and the actual shift of bandgap with temperature, which is induced by the ubiquitous radiative recombination of trap states. Therefore, for the sake of more accurate determination of bandgap shift, the bandgap energy was elaborately extracted through fitting the absorption coefficient near the band edge to the Elliot model. The extracted *E*_g_ value of CsPbBr_3_ 2‐ML NPLs exhibits an initial blueshift then redshift trend with temperature decrease, contrasting the monotonous redshift usually observed in CsPbBr_3_ bulk‐like NCs. Theoretical analyses based on the Bose–Einstein two‐oscillator model further uncovers that the more enhanced weight of contribution of electron‐optical phonon interaction in CsPbBr_3_ 2‐ML NPLs than in bulk‐like CsPbBr_3_ NCs is responsible for the blueshift‐redshift crossover of bandgap in the NPLs. Essentially, owing to the breaking translational periodicity in the thickness direction of 2D CsPbBr_3_ 2‐ML NPLs, the electron and phonon structures, and consequently the bandgap renormalization deriving from electron‐phonon interactions are apt to change remarkably relative to the quasi‐3D CsPbBr_3_ NCs counterparts. Meanwhile, the strong quantum confinement effect and the reduced dielectric screening due to the low dielectric constant of surface organic ligands in CsPbBr_3_ 2‐ML NPLs can also influence the electron‐phonon interactions. Therefore, we anticipate that, thinner CsPbBr_3_ NPLs are more likely to exhibit the observed unusual blueshift‐redshift crossover; the thinner the CsPbBr_3_ NPL is, the lower the critical temperature for the appearance of redshift trend becomes, and vice versa. The findings in this work provide new insights into the pivotal role of electron‐phonon interactions in the bandgap renormalization for 2D inorganic lead‐halide perovskites, which may pave the way for further investigations into the optical and optoelectronic properties of low‐dimensional perovskite nanomaterials.

## 4 Experimental Section

### Chemicals and Materials

Pb(CH_3_COO)_2_·3H_2_O (99.99%), Cs_2_CO_3_ (99.99%), HBr (48%), and trioctylphosphine (TOP, 98%) were purchased from Aladdin (Shanghai, China). Oleic acid (OA, 90%), oleylamine (OAm, 90%), and 1‐octadecene (ODE, 90%) were purchased from Sigma‐Aldrich (Shanghai, China). PbBr_2_ (99%) was purchased from Adamas. Dimethylformamide (DMF), hexane, toluene, and acetone were of analytical grade and purchased from Sinopharm Chemical Reagent Co. (Shanghai, China). All the chemical reagents were used as received without further purification.

### Synthesis of CsPbBr_3_ NCs

CsPbBr_3_ NCs were synthesized through a hot‐injection method by using HBr as the halide source to precipitate the perovskite QDs.^[^ ^9b^ ^]^ In a typical process of synthesizing CsPbBr_3_ NCs, 0.5 mmol of Pb(CH_3_COO)_2_·3H_2_O and 0.1 mmol of Cs_2_CO_3_ were mixed with 1 mL of OA, 1 mL of OAm, 6 mL of ODE and 1 mL of TOP in a 50 mL three‐neck round‐bottom flask. The obtained mixture was heated at 120 °C for 1 h under a N_2_ flow with constant stirring to form a clear solution and simultaneously remove residual water and oxygen. The temperature was then raised up to 180 °C and stabilized for 10 min, followed by rapid injection of 1.5 mmol of HBr into the hot solution. After 10 s, the reaction mixture was cooled down to room temperature by ice‐water bath. The resulting CsPbBr_3_ NCs were precipitated by addition of acetone, collected via centrifugation, washed with acetone and dispersed in hexane finally.

### Synthesis of CsPbBr_3_ 2‐ML NPLs

First, cesium oleate (Cs‐OA) precursor solution was prepared following the reported approach by Protesescu et al.^[^ ^9a^ ^]^ In a typical synthesis process, 0.35 g of Cs_2_CO_3_, 1.25 mL of OA and 20 mL of ODE were loaded into a three‐neck round‐bottom flask, then heated at 150 °C for 1 h under constant magnetic stirring in N_2_ atmosphere. Thereafter, 0.8 mL of PbBr_2_ precursor (0.735 mg of PbBr_2_ in 5 mL DMF) was swiftly injected into the mixture of 5 mL of ODE, 0.5 mL of OA, 0.5 mL of OAm, 60 µL of HBr, and 0.4 mL of as‐prepared Cs‐OA precursor.^[^ ^9c^ ^]^ After 10 s, 20 mL of acetone was added to quench the reaction. Finally, the obtained NPLs were precipitated by centrifugation and dispersed in toluene.

### Structural and Optical Characterization

XRD patterns of the samples were collected with an X‐ray diffractometer (MiniFlex2, Rigaku) with Cu K*α*1 radiation (*λ* = 0.154 187 nm). TEM measurements were performed using a FEI Tecnai F20 TEM. Absorption spectra were measured by using a tungsten lamp (Philips, 12 V, 10 W) as light source focused onto the round quartz substrate coated with sample by spinning, within a closed‐cycle liquid helium cryostat (CS202PE‐DMX‐1AL, 10–325 K). The signals of the transmitted light, when the sample was on or off the quartz substrate, were detected by a spectrometer (FLS980, Edinburgh). The emission spectra were recorded by replacing the tungsten lamp with continuous xenon lamp (450 W). All the spectra were recorded with a temperature interval of 20 K. For the photoluminescence (PL) decay measurement at 77 and 290 K, the sample was placed on a thermal stage (THMS 600, Linkam Scientific Instruments, 77–873 K) and excited by a 375‐nm ps‐pulsed laser and the signals were collected into a fiber bundle catheter, and detected by a FLS980 spectrometer. The absolute PL QYs of CsPbBr_3_ NCs dispersed in hexane and CsPbBr_3_ 2‐ML NPLs dispersed in toluene were measured by employing a standard barium sulfate coated integrating sphere (150 mm in diameter, Edinburgh) as the sample chamber that was mounted on the FLS980 spectrometer with the entry and output port of the sphere located in 90° geometry from each other in the plane of the spectrometer. A standard tungsten lamp was used to correct the optical response of the instrument. All the optical spectroscopic measurements were performed based on the NCs/NPLs thin films unless otherwise noted, and were corrected for the spectral response of both the spectrometer and the integrating sphere.

### Elliott Model

The absorption coefficient *α*(ℏ*ω*) near the band edge can be described by a modified Elliott model in the form:^[^ ^15b^ ^]^

(8) αℏω=AEbℏω∑m=1∞2Ebm3Γmℏω−Em2+Γm2∫Eg∞+∫Eg∞dE11−e−2πzΓcℏω−E2+Γc2

with *z* ^2^ = *E*_b_/(*E* − *E*_g_), *E*_m_ = *E*_g_ − *E*_b_/m^2^, m = 1, 2, 3..., Γ_m_ = Γ_c_ − (Γ_c_ − Γ_1_)/m^2^, where the first term represents the series of exciton resonance absorption with a Lorentzian line‐shape of linewidth Γ_m_ (*m* = 1, 2, 3…), and the second term denotes the absorption of continuum states convoluted with a Lorentzian function of linewidth Γ_c_, which can be solved by integration in the complex plane. Thus, the analytical expression is obtained:

(9a) $$\begin{array}{ccc} \alpha \left(\hbar \omega \right) & = & \frac{A\sqrt{E_{b}}}{\hbar \omega }\left\{\sum_{m=1}^{\infty }\frac{2E_{b}}{m^{3}}\frac{\Gamma_{m}}{{\left(\hbar \omega -E_{m}\right)}^{2}+\Gamma_{m}^{2}}\right.\\ & & +\frac{1}{2}\left[\frac{\pi }{2}+\arctan\left[\frac{\hbar \omega -E_{g}}{\Gamma_{c}}\right]\right]-\sum_{m=1}^{\infty }\frac{E_{b}}{m^{3}}\frac{\Gamma_{c}}{{\left(\hbar \omega -E_{m}\right)}^{2}+\Gamma_{c}^{2}}\\ & & \left.+\frac{\pi }{2}\frac{\sinh\left(2u^{+}\right)}{\cosh\left(2u^{+}\right)-\cos\left(2u^{-}\right)}\right\} \end{array}$$

with

(9b) $$u^{\pm }=\pi \sqrt{E_{b}/2}{\left[\frac{{\left[{\left(\hbar \omega -E_{g}\right)}^{2}+\Gamma_{c}^{2}\right]}^{1/2}\pm \left(\hbar \omega -E_{g}\right)}{{\left(\hbar \omega -E_{g}\right)}^{2}+\Gamma_{c}^{2}}\right]}^{1/2}$$

where *E*_m_ is the energy position of the *m* exciton line (*m* = 1, 2, 3…), *E*_b_ is the binding energy, and *E*_g_ is the bandgap energy. In this work, for simplicity, the difference between Γ_m_ and Γ_c_ was not taken into consideration, neither was the exciton line with *m* ≥ 2.

### The Physical Origin of Power‐Law Decay

Taking Several models have been developed to explain the physical origin of power‐law decay.^[^ ^21b^ ^]^ A common feature of such models is that charge‐carriers are localized in tail states (low‐lying trap states) with an occupation probability *p*(*t*) characterized with the form *p*(*t*) = *e* ^−*γt*^, where *γ* is the escape rate from a tail state to band edge. In Arrhenius detrapping model, the escape rate from a tail state of energetic depth *ε* to band edge has the form

(10) $$\gamma \left(\varepsilon \right)=\nu_{0}e^{-\varepsilon /K_{{}_{b}}T}$$

where *ν*_0_ is the attempt‐to‐escape frequency, *K*_b_ is Boltzmann constant. In the simplest Arrhenius detrapping model, the trapped charge‐carrier is assumed to rapidly undergo radiative recombination after escaping to the band edge. If *N*(*ε, t*) is the number of charge‐carrier in tail states of depth *ε* at time *t*, we assume that

(11) $$\frac{dN(\varepsilon ,t)}{dt}=-N\left(\varepsilon ,t\right)\gamma \left(\varepsilon \right)$$

Integrating then gives

(12) $$N\left(\varepsilon ,t\right)=N\left(\varepsilon ,0\right)exp\left(-\nu_{0}{e^{-}}^{\varepsilon /K_{b}T}t\right)$$

The PL intensity (*I*(*t*)) is proportional to the amount of supply of charge‐carriers to the band edge, so

(13) $$I\left(t\right)\propto -\int_{0}^{\infty }\frac{dN(\varepsilon ,t)}{dt}d\varepsilon$$

Thus, for an exponential energetic distribution of tail states with characteristic depth ɛ_0_, $n\left(\varepsilon \right)=Ae^{-\varepsilon /\varepsilon_{0}}$ and *N*(ɛ, 0)∝*n*(ɛ), then

(14) $$I\left(t\right)\propto \int_{0}^{\infty }Ae^{-\varepsilon /\varepsilon_{0}}\exp(-\nu_{0}e^{-\varepsilon /K_{b}T}t)\nu_{0}e^{-\varepsilon /K_{b}T}d\varepsilon$$

Substitution of $\varsigma =\nu_{0}e^{-\varepsilon /K_{b}T}t$ produces

(15) $$I\left(t\right)\propto A\frac{K_{b}T}{t}{\left(\nu_{0}t\right)}^{-K_{b}T/\varepsilon_{0}}\int_{0}^{\nu_{0}t}\varsigma^{K_{b}T/\varepsilon_{0}}e^{-\varsigma }d\varsigma$$

If *ν*_0_ *t* ≫ 1,

(16) $$I\left(t\right)\propto f\left(\nu_{0},K_{b}T/\varepsilon_{0}\right)t^{-(1+K_{b}T/\varepsilon_{0})}$$

## Conflict of Interest

The authors declare no conflict of interest.

## Supporting information

Supporting Information

## Data Availability

Research data are not shared.
