# Supplementary material for: Unusual Temperature Dependence of Bandgap in 2D Inorganic Lead‐Halide Perovskite Nanoplatelets
Source: Adv Sci (Weinh). 2021 Aug 11;8(19):2100084. doi: 10.1002/advs.202100084 (PMC8498867; doi:10.1002/advs.202100084)
Supplement: Supplementary file 1 — Supporting Information [file ADVS-8-2100084-s001.pdf]

© 2021 Wiley-VCH GmbH

ADVANCED SCIENCE Open Access

Supporting Information

for *Adv. Sci.*, DOI: 10.1002/advs.202100084

Unusual Temperature Dependence of Bandgap in 2D Inorganic Lead-Halide Perovskite Nanoplatelets

Shaohua Yu, Jin Xu, Xiaoying Shang, En Ma, Fulin Lin, Wei Zheng, Datao Tu, Renfu Li, and Xueyuan Chen**

Supporting Information

Unusual Temperature Dependence of Bandgap in 2D Inorganic Lead-Halide Perovskite Nanoplatelets

Shaohua Yu, Jin Xu,* Xiaoying Shang, En Ma, Fulin Lin, Wei Zheng, Datao Tu, Renfu Li, and Xueyuan Chen*

Supporting Tables

Table S1. Absolute PL quantum yields of $CsPbBr_3$ NCs and $CsPbBr_3$ 2-ML NPLs, and list of parameters derived from fitting the PL emissions of $CsPbBr_3$ NCs and 2-ML NPLs by the line-shape function.

$CsPbBr_3$	QY	W_d (eV)	E_a (eV)	E_g (eV)	N	M a)	A
**NCs**	73%	0.027	2.61	2.57	135	10000	1950
**2-ML NPLs**	18%	0.037	3.13	3.09	436	10000	1350

a) *M* was typically set to be 10000.[1]

Table S2. List of parameters derived from fitting the absorption coefficient near band edge by the Elliott model for $CsPbBr_3$ NCs over the temperature range of 10-290 K. The fitting parameter E_{1s} is the energy position of m = 1 exciton line, and Γ is the linewidth of Lorentzian function.

T (K)	E_g (eV)	E_b (meV)	E_{1s} (eV)	Γ (meV)	A
**290**	2.469	48	2.420	50	2.286
**270**	2.467	47	2.420	48	2.397
**250**	2.463	43	2.420	45	2.667
**230**	2.462	44	2.418	43	2.603
**210**	2.466	48	2.418	43	2.441
**190**	2.462	48	2.414	42	2.451
**170**	2.460	50	2.411	40	2.378
**150**	2.457	51	2.406	41	2.416
**130**	2.443	44	2.399	36	2.623
**110**	2.436	44	2.393	35	2.669
**90**	2.424	39	2.385	32	2.775
**70**	2.414	37	2.377	30	2.819
**50**	2.406	36	2.369	29	2.782
**30**	2.396	34	2.362	26	2.655
**10**	2.391	33	2.358	26	2.622

Table S3. List of parameters derived from fitting the absorption coefficient near band edge by the Elliott model for $CsPbBr_3$ 2-ML NPLs over the temperature range of 10-290 K.

T (K)	E_g (eV)	E_b[a)] (meV)	E_{1s} (eV)	Γ (meV)	A
**290**	3.119		2.889	45	1.120
**270**	3.125		2.895	47	1.334
**250**	3.134		2.904	41	1.019
**230**	3.137		2.907	40	1.308
**210**	3.140		2.910	41	1.302
**190**	3.140		2.910	33	0.957
**170**	3.140		2.910	35	1.249
**150**	3.140		2.910	34	1.235
**130**	3.139	230	2.909	32	1.251
**110**	3.139		2.909	31	1.283
**90**	3.137		2.907	31	1.270
**70**	3.136		2.906	29	1.298
**50**	3.134		2.904	29	1.284
**30**	3.131		2.901	28	1.263
**10**	3.128		2.898	27	1.207

a) E_b was approximately regarded as a constant independent of temperature variation when considering that $CsPbBr_3$ 2-ML NPLs exhibit strong quantum confinement and possess reduced dielectric screening due to the surface passivation of oleic acid ligand with low dielectric constant, thus largely enhancing the Coulomb interaction between electron and hole to form strongly bound exciton.[2]

Table S4. List of parameters derived from fitting the E_g values extracted from the absorption spectra over the temperature range of 10-290 K, for $CsPbBr_3$ NCs and 2-ML NPLs, respectively, by adopting the Bose-Einstein two-oscillator model.

$CsPbBr_3$	E_0 (meV)	E_{ac} (meV)	$A_{ac}/(M_{ac}E_{ac})$ (eV)	E_{opt} (meV)	$A_{opt}/(M_{opt}E_{opt})$ (eV)
**NCs**	2643.3	6.9	0.054	48.1	-0.557
**2-ML NPLs**	3313.93	4.0	0.007	55.0	-0.377

Table S5. List of parameters derived from fitting the full width at half maximum (FWHM) for $CsPbBr_3$ NCs and 2-ML NPLs, respectively, by adopting Segall's expression: $\gamma(T)=\gamma_0+A_{LO}/\left(e^{E_{LO}/K_bT}-1\right)$, where γ_0 is the temperature-independent zero-temperature linewidth arising from spectral diffusion, A_{LO} is the electron-optical phonon interaction coefficient, and E_{LO} represents the effective energy of optical phonon.

$CsPbBr_3$	γ_0 (meV)	A_{LO} (meV)	E_{LO} (meV)
**NCs**	15.80	54.25	14.87
**2-ML NPLs**	31.45	65.98	20.56

Supporting Figures

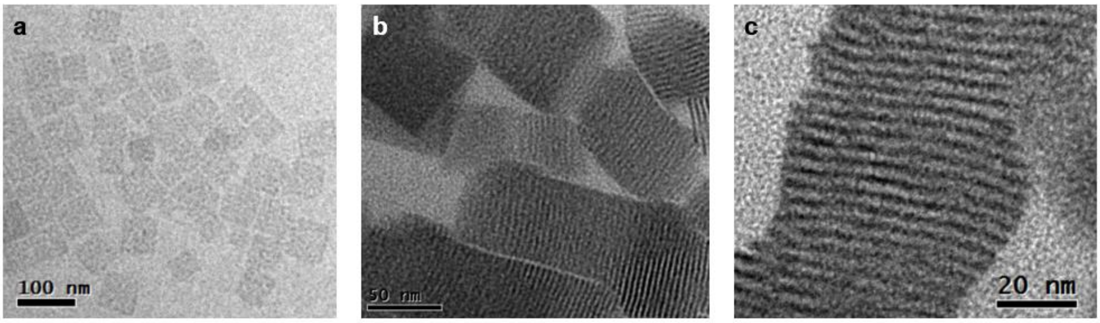

Figure S1. TEM images of $CsPbBr_3$ 2-ML NPLs flat-lying (a) and face-to-face stacking (b-c) on the copper grid.

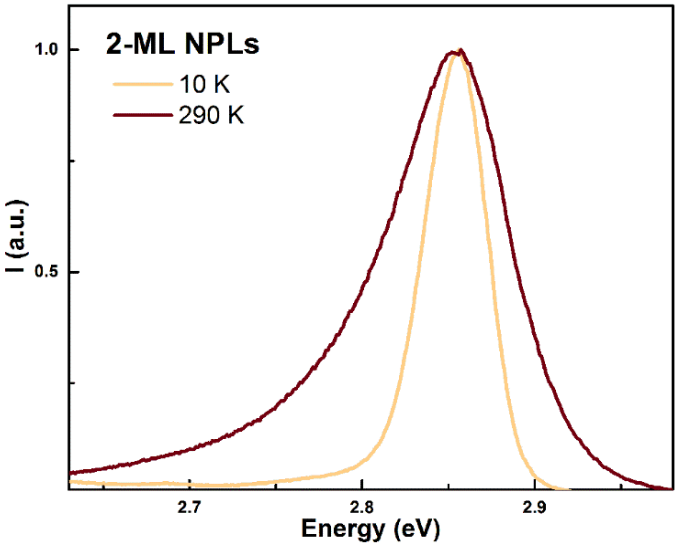

Figure S2. PL spectra of $CsPbBr_3$ 2-ML NPLs at 10 and 290 K, excited at 365 nm. The single PL peak at 10 K is indicative of the homogeneity in the thickness distribution of NPLs.

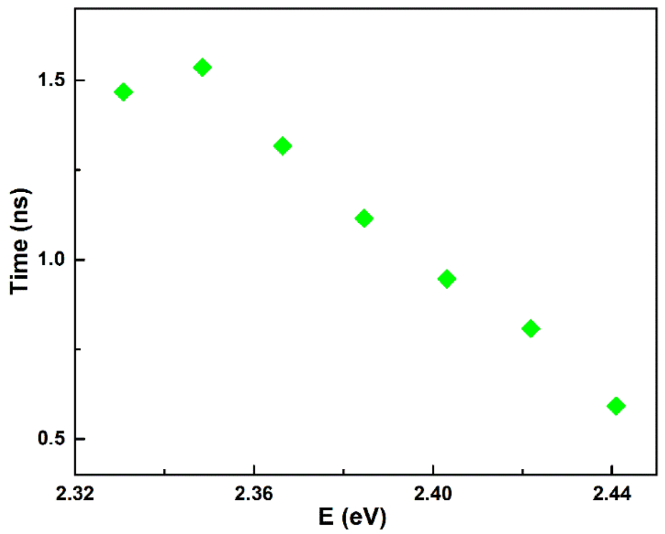

Figure S3. Wavelength-dependent PL lifetimes of $CsPbBr_3$ NCs at 77 K. The effective lifetime (τ_{eff}) is determined by: $\tau_{eff} = \frac{1}{I_{max}} \int_0^{\infty} I(t)\,dt$, where $I(t)$ denotes the PL intensity as a function of time, and I_{max} represents the maximum PL intensity.

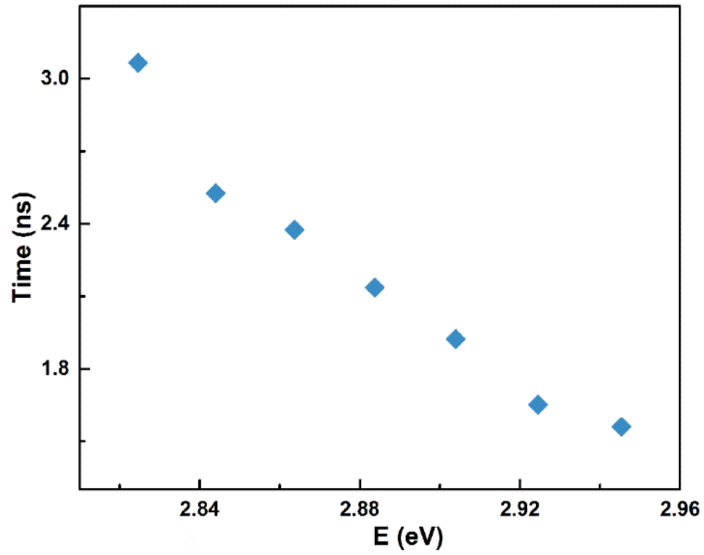

Figure S4. Wavelength-dependent PL lifetimes of $CsPbBr_3$ 2-ML NPLs at 77 K.

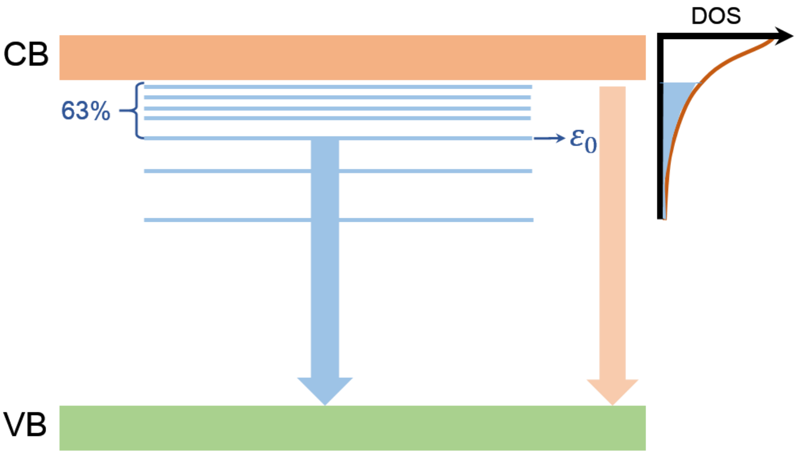

Figure S5. Schematic illustration of tail states (*i.e.*, low-lying trap states, as indicated by the series of blue lines) in $CsPbBr_3$ 2-ML NPLs. For an exponential energetic distribution of tail states (shadow area) with depth ε and characteristic depth ε_0, $n(\varepsilon) = A\mathrm{e}^{-\varepsilon/\varepsilon_0}$, where the ratio of traps in the tail states lying above ε_0 equals $\int_0^{\varepsilon_0} e^{-\varepsilon/\varepsilon_0} \mathrm{d}\varepsilon \Big/ \int_0^{\infty} e^{-\varepsilon/\varepsilon_0} \mathrm{d}\varepsilon = 1 - 1/e \approx 63\%$.

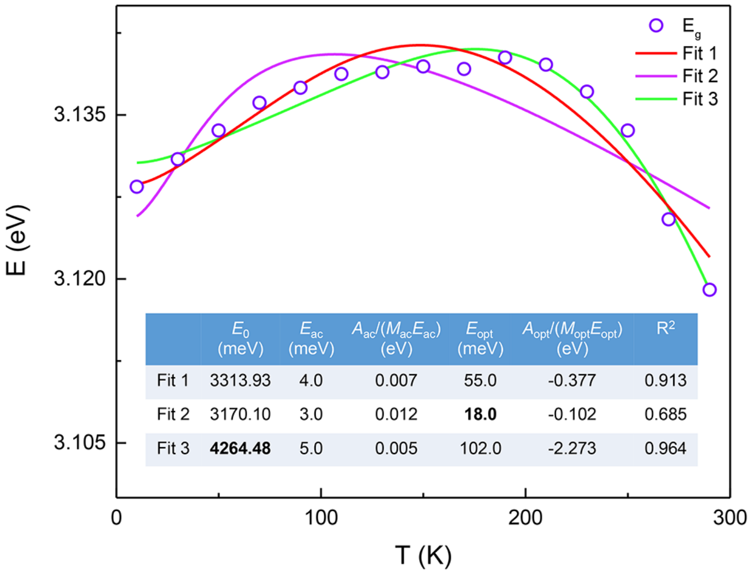

	E_0 (meV)	E_{ac} (meV)	$A_{ac}/(M_{ac}E_{ac})$ (eV)	E_{opt} (meV)	$A_{opt}/(M_{opt}E_{opt})$ (eV)	R^2
Fit 1	3313.93	4.0	0.007	55.0	-0.377	0.913
Fit 2	3170.10	3.0	0.012	**18.0**	-0.102	0.685
Fit 3	**4264.48**	5.0	0.005	102.0	-2.273	0.964

Figure S6. Representative fitting results of the temperature-dependent bandgap in $CsPbBr_3$ 2-ML NPLs by adopting the Bose-Einstein two-oscillator model. Inset table: list of the derived parameters for Fits 1-3.

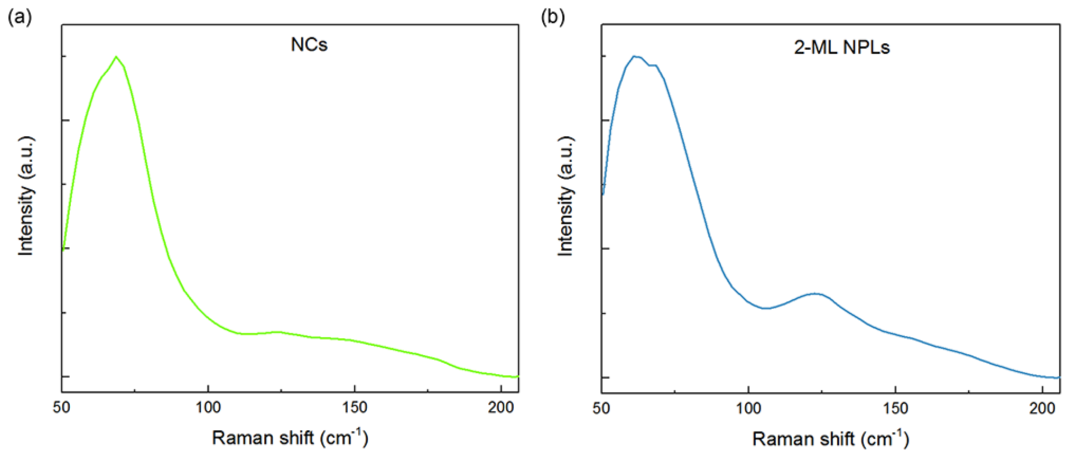

Figure S7. Raman spectra of $CsPbBr_3$ NCs (a) and 2-ML NPLs (b) measured in the range of 50-200 cm^{-1} at room temperature upon laser excitation at 633 nm. The maximum cut-off phonon energy of $CsPbBr_3$ crystal lattice is around ≈ 123 cm^{-1} (16 meV).[3]

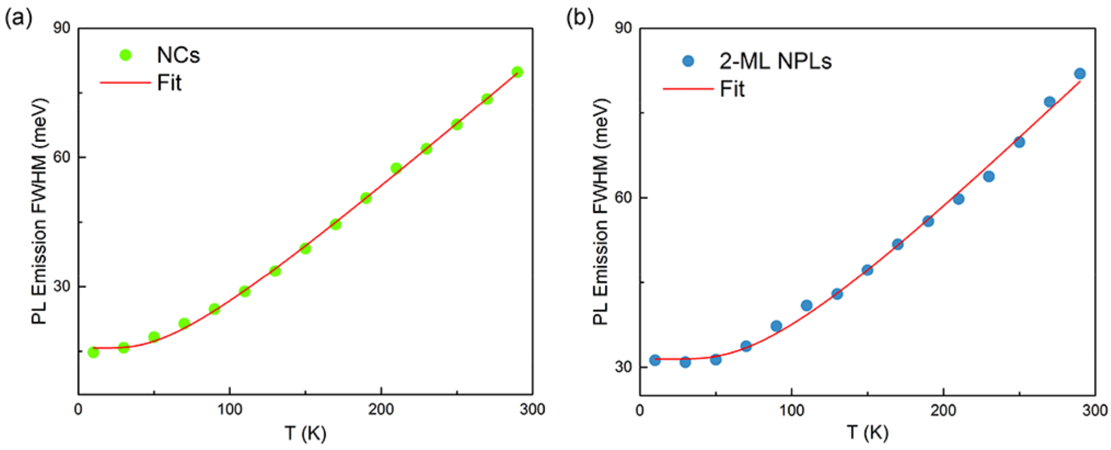

Figure S8. FWHM of PL emission spectra as a function of temperature for $CsPbBr_3$ NCs (a) and 2-ML NPLs (b), respectively. The red solid lines represent the fitting results based on Segall's expression: $\gamma(T) = \gamma_0 + A_{LO} / \left(e^{E_{LO}/K_b T} - 1\right)$, which takes into account the temperature-independent inhomogeneous broadening (γ_0) and the interaction between electron and longitudinal optical phonon.

References

[1] J. B. Gao, J. C. Johnson, *ACS Nano* **2012**, *6*, 3292-3303.

[2] H. Hiraga, T. Makino, T. Fukumura, A. Ohtomo, M. Kawasaki, *Appl. Phys. Lett.* **2009**, *95*, 211908.

[3] S. Muduli, P. Pandey, G. Devatha, R. Babar, Thripuranthaka M, D. C. Kothari, M. Kabir, P. Pillai, S. Ogale, *Angew. Chem. Int. Ed.* **2018**, *57*, 7682-7686.
